# Sufentanil EC50 for endotracheal intubation with aerosol inhalation of carbonated lidocaine by ultrasonic atomizer

**DOI:** 10.1186/s12871-021-01367-w

**Published:** 2021-05-12

**Authors:** Qiaoqiao Xu, Zhiqiang Zhou, Ling Ai, Jieqiong Liu, Xuebi Tian

**Affiliations:** Department of Anesthesiology, Tongji Hospital, Tongji Medical College, Huazhong University of Science and Technology, 1095 Jie Fang Avenue, Wuhan, 430030 Hubei China

**Keywords:** Aerosol inhalation, Lidocaine, Endotracheal intubation, EC50, Sufentanil

## Abstract

**Background:**

Nebulized lidocaine reduced stress response for endotracheal intubation. However, the impact of novel lidocaine aerosol inhalation for intubation by ultrasonic atomizer was unclear. Hence, we designed aerosol inhalation of lidocaine by ultrasonic atomizer, to seek whether the dosage of sufentanil for intubation could be less or not.

**Methods:**

Intravenous injection of sufentanil started at 0.5 μg/kg, and sufentanil dosage was increased/decreased (step-size 0.05 μg/kg for sufentanil) using Dixon’s up and down method. The observation was terminated after 8 reflexes.

**Results:**

The EC50 and EC95 of sufentanil with lidocaine by ultrasonic atomizer for intubation were found to be 0.232 μg/kg (95% CI: 0.187–0.270 μg/kg) and 0.447 μg/kg (95% CI: 0.364–0.703 μg/kg). 55.88% out of 34 patients showed hemodynamic index change < 20% of baseline during intubation.

**Conclusion:**

Aerosol inhalation of lidocaine by ultrasonic atomizer reduced the dosage of sufentanil for endotracheal intubation. Lidocaine inhalation by ultrasonic atomizer for airway anesthesia with minimal dosage of sufentanil could be recommended, particularly in patients who need more stable hemodynamic changes or spontaneous respiration.

**Trial registration:**

Chinese Registry of Central Trial, ChiCTR-IOR-17014198.

Registered 28 December 2017.

## Background

Endotracheal intubation was a routine procedure in clinical general anesthesia. But the placement of laryngoscope and endotracheal tube irritated the glottis and trachea leading to the reflex sympathetic reaction [1]. There were several ways to reduce the stress response and attenuate the adverse hemodynamics fluctuations during laryngoscopy placement and intubation, such as enhancing the depth of anesthesia with high dosage of opioids, airway surface anesthesia with local anesthetic, or negative inotropic drugs and antihypertensive drugs to prevent the adverse cardiovascular events. High dosage of opioids caused side effects like respiratory depression, hemodynamic fluctuation and intestinal peristalsis decrease [2]. Cardiovascular drugs were high risk factors for cardiovascular and cerebrovascular diseases in elder [3, 4]. Therefore, slowly anesthesia induction, gently intubation and low-opioid anesthesia were suggested in patients, particularly with various complications.

However, endotracheal intubation could not be carried out with no stress response under opioid-free anesthesia. Lidocaine was often used for oral airway surface anesthesia. Topical lidocaine surface anesthesia combined with minimal opioid was recommended in awake tracheal intubation [5]. Both 2 and 4% lidocaine administered topically could provide clinically acceptable intubating conditions for awake intubation [6, 7]. Airway lidocaine had a rapid onset of action (1–5 min) and intermediate duration of efficacy (10–15 min) [8]. The traditional administration was just spray lidocaine directly onto part of airway mucosa, the uncomfortable, hypertension or heart rate increase in patients were still existed. The lidocaine inhalation for airway anesthesia by ultrasonic atomizer was easy and valid. Evidence showed inhalation of lidocaine attenuated the response to airway irritation with lower plasma concentrations [9].

The lidocaine inhalation by ultrasonic atomizer combined with minimal opioid could be optimized strategic for the elderly patients. Meanwhile, it was practical for these special patients with difficult airway, who need less opioid to maintain spontaneous respiration. Moreover, these patients were suitable to receive lidocaine inhalation, who were required spontaneous respiration by laryngeal mask with low-opioid anesthesia, like thoracoscopic surgery. However, the effective concentration of the minimal opioid was indistinct. Therefore, we speculated that aerosol inhalation of carbonated lidocaine could reduce the amount of sufentanil used. And, in this study, we aimed to investigate the sufentanil EC50 for endotracheal intubation with inhalation lidocaine by ultrasonic atomizer.

## Methods

### Inclusion and exclusion criteria

Our study was a single centre, prospective, double-blind clinical trial. The EC50 of sufentanil for endotracheal intubation was estimated with aerosol inhalation of nebulized lidocaine.

Testing was performed in Tongji Hospital, Tongji Medical College, Huazhong University of Science and Technology, 1095 Jie Fang Avenue, Wuhan 430,030, Hubei, China. Patients, American Society of Anesthesiology (ASA) physical status I or II, age 18–70 years, BMI < 25 without difficult airway, were scheduled to undergo elective surgery (ophthalmology or gynecology). The hepatic function, renal function, and ECG results were normal in these patients. With no liver and renal dysfunction and no cardiovascular disease. Patients eventually enrolled were 23–63 years old. The one was excluded with a history of local anesthetic allergy, oral and otolaryngologic lesions or surgical history, cardiovascular disease and COPD (chronic obstructive pulmonary disease). Patients in other clinical trials or researchers were excluded. All patients were given informed consent, and signed the informed consent form. They were evaluated preoperatively. A complete history of present and previous illness was taken, general physical examination and systemic examination were conducted to assess the fitness for the proposed procedure. Premedication was not prescribed to any patients.

### Study protocol

The Dixon’s up and down method was adopted [10], and intravenous injection of sufentanil starting at 0.5 μg/kg. Then sufentanil dosages were increased/decreased (step-size 0.05 μg/kg for sufentanil) using Dixon’s up and down method in the next patient, depending upon the previous patient’s response within 3 min after intubation. If the hemodynamic index change < 20% of baseline during endotracheal intubation, the sufentanil dosage would be decreased 0.05 μg/kg in next patients. If the hemodynamic index change > 20% of baseline during endotracheal intubation, the sufentanil dosage would be increased 0.05 μg/kg in next patients. The decrease to increase point, or the increase to decrease point of sufentanil dosage were as one of the reflexes. The observation was terminated after 8 reflexes. Endotracheal intubation was performed and scored by the same attending physician.

Forty patients were screened for eligibility, monitored by blood pressure (BP), pulse (P), electrocardiogram (ECG), SpO_2_, Nacrotrend values and muscle relaxant monitoring (train of four, TOF) during perioperative period. Thirty-six eligible patients were recruited in the study, 19 males and 17 females. After intravenous cannulation, Allen’s test was performed routinely before radial arterial artery cannulation, to make sure the puncture could be carried out without severe complications. The radial artery puncture and catheter under local anesthesia were established for arterial blood pressure (ABP) monitoring. Aerosol inhalation of 4 mg/kg carbonated lidocaine by the ultrasonic atomizer (YUWELL, 402B) was accomplished, prior to induction of anesthesia with a special atomizing nozzle. The atomizing nozzle was in the patient’s mouth airtightly. Waited 5 min for lidocaine to take effect. Then intravenous of anesthesia induction was achieved with propofol TCI (according to Nacrotrend monitoring anesthesia depth), rocuronium 0.9 mg/kg, and sufentanil. Until Nacrotrend value to 40 and TOF value to 0, endotracheal intubation was implemented by the experienced anesthesiologist using standard Macintosh blade laryngoscope. After that during maintenance of anesthesia, propofol and remifentanil were pumped continuously, and rocuronium was injected intermittently.

The time points needed record were those: prior to the aerosol lidocaine inhalation (T0), after the aerosol lidocaine inhalation (T1), after intravenous induction (T2), at the time point of endotracheal intubation (T3), and 1 min (T4), 2 min (T5), 3 min (T6) after endotracheal intubation. HR, P, BP, SpO_2_, and Nacrotrend values were collected.

### Blinding

The observations of response to endotracheal intubation were recorded by an independent anesthetist. Monitor screen was applied between the anesthetist who was responsible for observations and the anesthetist who managed endotracheal intubation. The patients and the anesthetists for intubation were not aware of the dosage of sufentanil. The sufentanil administered by another superior anesthesiologist, who calculated the dosage of sufentanil according the previous patient’s response within 3 min after intubation.

### Outcome measures

The primary outcome was effective concentration (EC50) of sufentanil causing “hemodynamic index change < 20% of baseline” during endotracheal intubation in 50% of study population. Adverse events to circulation (HR, BP) were noted as secondary outcomes.

### Statistical analysis

Statistical analysis was performed using Excel 2007 (Microsoft, Redmond, WA, USA) and SPSS version 15.0 software (IBM, Armonk, NY, USA). Patients’ characteristics were presented as mean (SD) or absolute numbers (percentages). Continuous variables were analyzed by t-test and categorical variables were analyzed by χ^2^ test. Sufentanil EC50 was calculated by modified Dixon’s up and down method (MDUDM) [11]. The mean of mid-point of all unsuccessful/successful pairs was used to determine EC50 using Dixon’s up and down method. Dose–response curve for EC50 with 95% confidence intervals (CI) were determined using probit regression analysis. Sample size was calculated based on the fact that a minimum of 8 crossover pairs were required for the analysis. The Pearson correlation analysis was used to study the correlation between patient’s characteristics and response to endotracheal intubation.

## Results

**Fig. 1:**
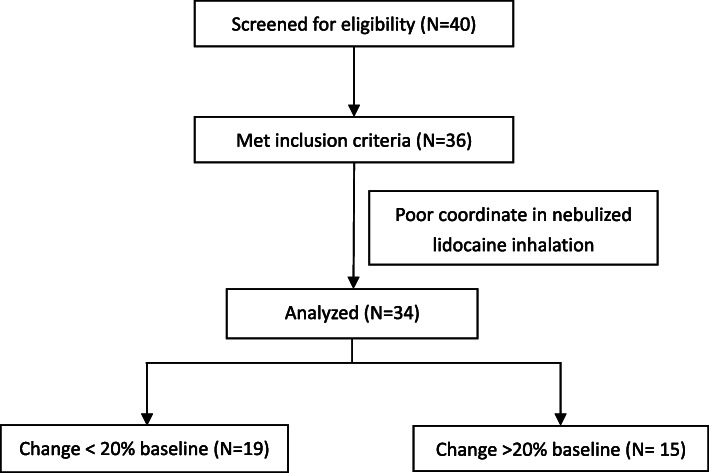
Study flow diagram

We assessed 40 patients for eligibility. Thirty-six eligible patients were recruited in the study (Fig. 1). Two patients were excluded from the study because of the poor coordinate in lidocaine inhalation. Demographic characteristics of study population were presented in Table 1.

**Table 1:** Demographic data of study population

Male/Female	16/18
ASA I/II	29/5
Age (y)	47.35 ± 11.87
Height (cm)	162.65 ± 7.08
Bodyweight (kg)	58.15 ± 6.76
BMI	22.00 ± 2.32
Mallampati Grade I/II	31/3

Values are presented as mean ± SD or absolute numbers

**Fig. 2:**
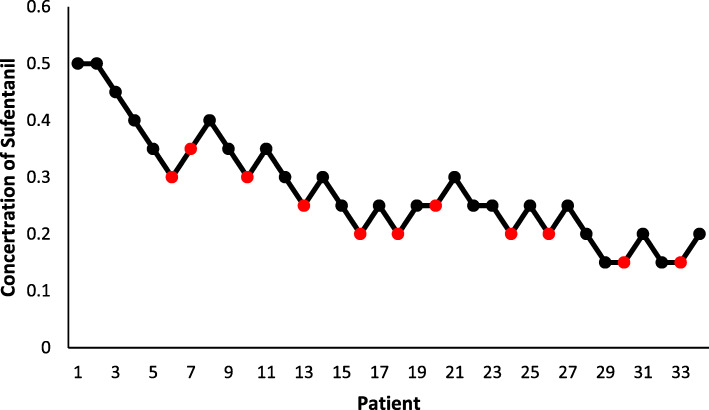
The 34 consecutive patients were attempted, and the concentration of sufentanil was determined according to the Dixon’s up-and-down method

**Fig. 3:**
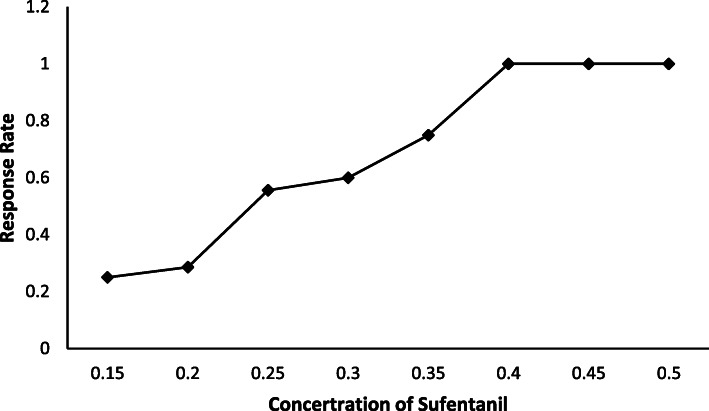
Concentration response curve of sufentanil and the respective reactions to endotracheal intubation was plotted from probit analysis

The EC50 and EC95 of sufentanil with aerosol inhalation of lidocaine for endotracheal intubation were found to be 0.232 μg/kg (95% CI: 0.187–0.270 μg/kg) and 0.447 μg/kg (95% CI: 0.364–0.703 μg/kg). The intravenous dosage of sufentanil-response data obtained by the up-down method (Fig. 2). The concentration of sufentanil and the response curve to endotracheal intubation were presented in Fig. 3.

**Fig. 4:**
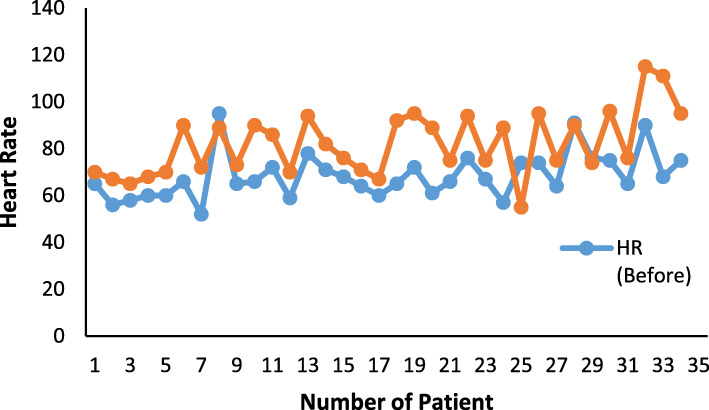
The heart rate values of 34 patients before and after endotracheal intubation were shown

55.88% out of 34 patients showed hemodynamic index change < 20% of baseline during endotracheal intubation. Fifteen patients (44.12%) showed hemodynamic index change > 20% of baseline during endotracheal intubation. None of the patient showed laryngospasm, local anesthetic allergy, sore throat, or hoarseness. The amplitude of hemodynamic indexes (HR and BP) variation for each patient were shown in Fig. 4.

## Discussion

Therapeutic uses of nebulized lidocaine for the upper airway were verified. Evidences showed the therapeutic uses of nebulized lidocaine in the treatment of intractable cough, asthma and reactive airway dysfunction syndrome [12–14]. Nielson et al. prospectively evaluated the effect of topical lidocaine in children, and they found lidocaine exaggerated findings associated with laryngomalacia that resulted in a higher laryngomalacia score [15]. Inhalation of lidocaine attenuated the response to airway irritation with plasma concentrations lower than those systemic administration [9, 16]. Topical lidocaine reduced upper airway reflexes (cough, mechanoreceptor reflexes, and genioglossus muscle activity) and impaired the arousal response [17–19].

Koirala S reported a case about topical anesthesia of the vocal cords by nebulized lidocaine inhalation, to facilitate fiberoptic nasotracheal intubation in a head-size parotid tumor patient, emphasizing the possibility of fiberoptic intubation in a sedated yet spontaneously breathing patient by allowing inhalation of nebulized lidocaine [20]. However, only lidocaine through ultrasonic nebulizer may not provide acceptable conditions for awake fiberoptic bronchoscopy [21]. And then, nebulized lidocaine combined with fentanyl, as a premedication to general anesthesia, was a recommended implementation in spontaneously breathing patients undergoing rigid bronchoscopy [22]. It was also recommended the cautious use of minimal sedation and analgesia, and combined encouraged appropriate local anesthetic topicalization in awake tracheal intubation [5]. Opioids, administered as bolus or continuous infusion at the reported dosages, appeared safe and effective with some advantage in reducing coughing and gag reflex [23]. A minimal opioids technique could be safely and effectively performed to reduce patient anxiety, discomfort, and increased patient co-operation level [24, 25].

There were several topical lidocaine administration strategies for upper airway as follows [26]: the patient gargled 2% viscous lidocaine while positioned upright, administered using a small disposable drinking cup; an alternative to the previous step involved the use of lidocaine paste; some clinicians used lidocaine-soaked pledgets as part of the procedure; 4% lidocaine was administrated to oropharyngeal and glottic structures using an oxygen-driven power sprayer; 4% lidocaine was administered through the airway guide using special device; the translaryngeal block numbed the larynx and trachea with injected lidocaine through the cricothyroid membrane, inducing coughing that scattered the local anesthetic; nerve blocks by lidocaine injection subdermal for superior laryngeal nerve and recurrent laryngeal nerve block. The effectivity and safety of all these topical lidocaine administration strategies needed to be sure. Nebulization of lidocaine by a device for intermittent positive pressure breathing (IPPB 280 mg) or by an ultrasonic (400 mg), nebulizer was reported as a topical anesthetic for the airway, with favorable results [27]. And aerosol inhalation of nebulized lidocaine local anesthesia by atomization device could provide a more comprehensive, convenient and fast way for Oral-Laryngo-Tracheal omnibearing mucosal contact before intubation.

For the effect on regional deposition, the significant of particle aerodynamic diameter and inhalation maneuver needed to be concerned [28]. Large particles (5–15 μm) were mainly deposited in the upper airways and trachea, intermediate-sized particles (3–5 μm) were tendency deposited in the bronchi and bronchioles, and small particles (≤ 3 μm) were flowed into the alveoli [29]. The aerosol characteristics were closely related to the device of atomizer, which depended on the design of pressure swirl, airblast and ultrasonic atomizers [30]. The ultrasonic atomizers operated at different frequencies, which were well with the median droplet size [31]. The piezoelectric part of the ultrasonic atomizer (YUWELL, 402B) produced 35% carbonated lidocaine particle sizes above 5.0 μm, which providing good surface analgesic effect in upper airway and trachea. The maximum rate of aerosol inhalation was more than 3.0 ml/min. And it just took 10 min to prepare and finish the whole process of inhalation.

Aerosol inhalation of nebulized lidocaine local anesthesia caused fewer trauma to the oropharyngeal and laryngeal tissues, avoided the risk of injection into vessels compared with nerve blocks, and possibly decreased the risk of systemic toxicity [32]. Lidocaine plasma concentrations below 6.0 μg/ml were considered safe. Given the lidocaine was administered by infiltration, the occurrence of neurological symptoms in healthy volunteers was about 8 mg/kg, corresponding to a plasma value of about 15 μg/ml [33]. The lidocaine plasma concentration was 0.7 ± 0.3 μg/ml when inhalation of lidocaine was 5 mg/kg [34]. In our study, the time of atomization inhalation lasted 5 min, and the dose of lidocaine was about 300 mg, it was safe as lidocaine plasma concentrations was far below 6.0 μg/ml.

In addition, we used carbonated lidocaine for inhalation in our study. The reason was that surface anesthetic effect of carbonated lidocaine was 4 times more than lidocaine hydrochloride [35]. Because the carbon dioxide released following permeation could produce local vasodilatation which increased the rate of absorption. In addition, as the carbon dioxide released following permeation, there was a resultant increase in pH, which augmented formation of free base. In that way, the local anesthetic was readily diffused across biological membranes, and the neural and vascular uptake were facilitated [36].

By Adamus M, excellent intubation conditions were observed in 28, 41 and 54%, while poor conditions were present in 31, 7 and 3% of patients each receiving sufentanil 0.2, 0.3 or 0.4 μg/kg respectively. Therefore, sufentanil (0.3–0.4 μg/kg in combination with propofol (2 mg/kg) provided clinically acceptable intubating conditions in 93–97% patients [37]. In our study, inhalation aerosol lidocaine was accomplished prior to induction of anesthesia. The intravenous of anesthesia was achieved with propofol, rocuronium 0.9 mg/kg, and combined with different adjusting dosage of sufentanil according to the reaction for intubation starting at 0.5 μg /kg. However, it was indicated that inhalation aerosol lidocaine reduced the amount of sufentanil needed for endotracheal intubation, and the EC50 sufentanil with aerosol lidocaine was only found to be 0.232 μg/kg. Twenty-three (55.88%) out of the all 34 patients showed hemodynamic index change < 20% of baseline during endotracheal intubation. It signified that combined with inhalation aerosol lidocaine for endotracheal intubation reduced the dosage of opioids, enhanced hemodynamic stability, and provided better intubation conditions.

A major limitation of this study was that each patient routinely received lidocaine for a fixed period of time (5 min) with aerosol inhalation, not according to the patient’s individual differences, which might impact the results. Moreover, only 35% particles of aerosol inhalation were the ideal size for the upper airways. A more efficient method or medical facilities of aerosol inhalation for upper airway surface anesthesia would be still worthy of further exploration.

## Conclusion

The advantages in carbonated lidocaine inhalation by ultrasonic atomizer for airway anesthesia were revealed in our study. Inhalation of aerosol carbonated lidocaine was expected to reduce the amount of sufentanil obviously, and then provided stable hemodynamic change which avoiding or reducing the usage of cardiovascular drugs. Therefore, for the patients with cardiovascular and cerebrovascular diseases, especially in elders need low-opioid, the aerosol inhalation of lidocaine by the ultrasonic atomizer before endotracheal intubation was the key step in anesthesia induction. The patients, who needed awake tracheal intubation as difficult airway, were provided a more effective and mucosal all-sided airway anesthesia technique with lower sufentanil dosage to keep spontaneous respiration. And in some natural airway video assisted thoracoscopic surgery in ERAS (Enhanced Recovery After Surgery), which reserved the spontaneous respiration without intubation, the patient was prepared and managed the intubation in case. Under the spontaneous respiration, the pulmonary tissue of the operation side could only be flat under the atmospheric pressure. The aerosol inhalation of lidocaine by the ultrasonic atomizer was a suitable method to prepare the airway for intubation in these cases as well, which required the minimal amount of sufentanil to make sure the spontaneous respiration recovery as soon as possible, and the spontaneous respiration was maintained in the whole surgery. However, the intubation could be implemented as prearranged under upper airway surface anesthesia by inhalation aerosol lidocaine at any emergency situation. Hence, there were more clinical prospects with aerosol inhalation of carbonated lidocaine by ultrasonic atomizer, such as awake endotracheal intubation, and so on.

## Data Availability

The data of this article was available from the corresponding author. The email address of the corresponding author was tianxb@hust.edu.cn.

## Abbreviations

ABP: Arterial blood pressure
ASA: American society of anesthesiology
BP: Blood pressure
CI: Confidence interval
COPD: Chronic obstructive pulmonary disease
EC50: Concentration for 50% of maximal effect
ECG: Electrocardiogram
HR: Heart rate
P: Pulse
SpO_2_: Pulse oxygen saturation
TCI: Target control infusion
TOF: Train of four
ERAS: Enhanced recovery after surgery
